# Outcomes following percutaneous coronary intervention and coronary artery bypass grafting surgery in Chinese, South Asian and white patients with acute myocardial infarction: administrative data analysis

**DOI:** 10.1186/1471-2261-13-121

**Published:** 2013-12-26

**Authors:** Danijela Gasevic, Nadia A Khan, Hong Qian, Shahzad Karim, Gerald Simkus, Hude Quan, Martha H Mackay, Blair J O’Neill, Amir F Ayyobi

**Affiliations:** 1 Cardiac Services, Royal Columbian Hospital, New Westminster, BC, Canada; 2 Division of General Internal Medicine, St. Paul’s Hospital, Vancouver, BC, Canada; 3 Centre for Health Evaluation and Outcome Sciences, St. Paul’s Hospital, 1081 Burrard Street, room 620-B, Vancouver, BC V6Z 1Y6, Canada; 4 Department of Medicine, University of Calgary, Calgary, AB, Canada; 5 School of Nursing, University of British Columbia, Vancouver, BC, Canada; 6 Heart Centre, St. Paul’s Hospital, Vancouver, BC, Canada; 7 Department of Medicine, Division of Cardiology, University of Alberta, Edmonton, Canada

**Keywords:** PCI, CABG, Ethnicity, AMI, Outcomes

## Abstract

**Background:**

Little is known on whether there are ethnic differences in outcomes following percutaneous coronary intervention (PCI) and coronary artery bypass grafting surgery (CABG) after acute myocardial infarction (AMI). We compared 30-day and long-term mortality, recurrent AMI, and congestive heart failure in South Asian, Chinese and White patients with AMI who underwent PCI and CABG.

**Methods:**

Hospital administrative data in British Columbia (BC), Canada were linked to the BC Cardiac Registry to identify all patients with AMI who underwent PCI (n = 4729) or CABG (n = 1687) (1999–2003). Ethnicity was determined from validated surname algorithms. Logistic regression for 30-day mortality and Cox proportional-hazards models were adjusted for age, sex, socio-economic status, severity of coronary disease, comorbid conditions, time from AMI to a revascularization procedure and distance to the nearest hospital.

**Results:**

Following PCI, Chinese had higher short-term mortality (Odds Ratio (OR): 2.36, 95% CI: 1.12-5.00; p = 0.02), and South Asians had a higher risk for recurrent AMI (OR: 1.34, 95% CI: 1.08-1.67, p = 0.007) and heart failure (OR 1.81, 95% CI: 1.00-3.29, p = 0.05) compared to White patients. Risk of heart failure was higher in South Asian patients who underwent CABG compared to White patients (OR (95% CI) = 2.06 (0.92-4.61), p = 0.08). There were no significant differences in mortality following CABG between groups.

**Conclusions:**

Chinese and South Asian patients with AMI and PCI or CABG had worse outcomes compared to their White counterparts. Further studies are needed to confirm these findings and investigate potential underlying causes.

## Background

Coronary revascularization including percutaneous coronary intervention (PCI) and coronary artery bypass graft (CABG) surgery, is among the most common and most expensive major medical procedures provided in North America [1], and is recommended by national guideline bodies for the management of selected patients with acute myocardial infarction (AMI) [2,3]. Percutaneous coronary intervention has been shown to be more effective than thrombolytic therapy for reperfusion [2,4]. In patients with AMI whose coronary anatomy appears unsuitable for PCI, CABG [3] or conservative treatment is indicated.

As burden of coronary heart disease has been increasing in developing countries such as India and China [5,6], use of coronary revascularization technology will likely rise globally. However, there is little evidence on the effectiveness of these procedures in South Asian and Chinese populations with AMI and how it compares to White population. Although no studies explored the ethnic differences in outcomes in the post-AMI post-revascularization setting, in patients with coronary artery disease who have undergone revascularization procedures, studies yielded conflicting results. Namely, while some studies reported no difference in mortality rates following PCI between South Asian and White patients [7,8], other observed lower mortality among Asian patients compared to their Western European counterparts [9]. Similarly, among studies exploring the ethnic differences in fatal cardiac outcomes following CABG, some studies reported higher mortality in South Asian compared to White patients [10-12], while others observed no such difference [13]. However, these studies are limited by lack of adjustment for differences in baseline prognostic characteristics, some by small sample sizes, and the effectiveness of revascularization is known to differ between AMI populations and those with stable coronary artery disease [2,14]. Therefore, the objective of this analysis was to evaluate ethnic differences in fatal and non-fatal outcomes following PCI and CABG in Chinese, South Asian, and White patients who have suffered acute myocardial infarction (AMI) while controlling the analyses for potential confounders such as socio-demographics, severity of coronary artery disease, and presence of comorbid conditions.

## Methods

### Data sources

For this retrospective cohort study, data were derived from the hospital administrative data collected in the province of British Columbia (BC), Canada, and the BC Cardiac Registry from April 1999 to March 2003. The hospital administrative data include demographics, admission and discharge dates, and most responsible diagnosis for admission along with comorbid conditions for all patients admitted to hospital in BC. The BC Cardiac Registry data include the type, date and clinical details of the revascularization procedure performed, data on the indication for the procedure, extent of coronary artery disease, complications and other outcomes. The Registry includes all such procedures performed in the province. The Vital Statistics file contains the date of death for all patients with legislated mandatory reporting and is updated daily. Data were linked using a unique identifier common to all databases.

### Study population

All patients with AMI diagnosed 1999–2003 in BC were identified using a validated coding algorithm in the hospital administrative database [15] based on the most responsible diagnosis code (*International Classification of Diseases*, ninth revision [ICD 9], 410.x). This coding algorithm has a positive predictive value of 95% (91% to 98%) identifying the physician’s diagnosis of acute myocardial infarction from hospital charts [16]. Patients younger than 20 years of age and those who were discharged from hospitalized for less than one day were excluded from the study to reduce the risk of false-positive diagnosis of AMI.

### Revascularization and outcomes

For the study primary end-points, short (30-day) and long-term mortality, date of death was obtained from the BC Vital Statistics file. The secondary end-points included first recurrence of AMI and readmission to the hospital for heart failure (ICD 9, 428.x), and these were derived from hospital administrative data. For the study end-points, patients were followed-up for a maximum of 4 years.

### Other prognostic variables

To adjust for baseline prognostic differences between groups, we used the mortality prediction rule following AMI [17] that was validated for 30-day and 1-year mortality displaying reasonable accuracy (areas under the curve was 0.78 and 0.79 for 30-day and 1-year mortality, respectively). The variables included age (dichotomized into 20–64 and 65 years of age and older), sex, area-level socio-economic status (assessed from area-level median household income based on the 2001 Canadian Census), and presence of comorbid conditions such as heart failure, cardiogenic shock, arrhythmia (any atrial or ventricular arrhythmia), diabetes mellitus, cerebrovascular disease, cancer, and acute or chronic renal disease. We also determined distance to the nearest hospital (defined as distance between the postal code centroids of the residential area and the nearest hospital), duration from diagnosis of AMI to revascularization procedure examined in the model, and severity of coronary artery disease based on Duke Criteria [18,19].

### Defining ethnicity

Ethnicity data were not available in administrative data. The Nam Pehchan computer program [20] and Chinese surname list [21] were used to identify study participants of South Asian and Chinese ethnicity, respectively. According to the 2006 Canadian Census, in British Columbia Canadian province, South Asians and Chinese were the two largest visible minority groups, while the remaining population was predominantly White [22]. Consequently, the rest of the study non-South Asian and non-Chinese participants were classified as White. Nam Pehchan program has been shown to classify South Asian names with 90.5% sensitivity and 99.4% specificity, while a positive predictive value of the program was 63.2% [20]. Sensitivity, specificity and a positive predictive value of the Chinese surname list are 77.7%, 99.7%, and 80.5% respectively [21]. In the event of changing surnames following an interracial marriage, misclassification may occur; however, using Chinese surname list, there was only a small drop in sensitivity when married females (Sensitivity = 73.2%) were compared to never-married ones (76.7%). In addition, validation of Nam Pehchan program revealed only 0.05% of the 356,555 names to have mixed components of South Asian and non-South Asian origins [20].

### Statistical analysis

Ethnic differences in baseline characteristics were tested using Chi-square test or Fisher’s exact test, where appropriate. Ethnic differences in short-term mortality (up to 30 days after AMI) were modeled using logistic regression. Furthermore, ethnic differences in long-term mortality (30 days to a maximum of 4 years after index AMI), time to the first recurrent AMI, and hospitalization for heart failure were evaluated using Cox proportional-hazards modeling. The proportional hazard assumption was assessed using the methods based on cumulative sums of martingale residuals [23]. Firth correction methods were applied in the regression models in case of monotone likelihood with infinite estimates [24,25]. All regression models were adjusted for age, sex, socio-economic status, distance to the nearest hospital, duration from the diagnosis of AMI to a revascularization procedure examined in the model, severity of coronary disease and presence of comorbid conditions (congestive heart failure, cardiogenic shock, arrhythmia, diabetes mellitus, cerebrovascular disease, cancer, and acute or chronic renal disease) based on the Ontario AMI mortality prediction rule [17]. Analyses were performed using SAS statistical software version 9.1 (SAS Institute Inc., Cary, NC). The University of British Columbia institutional research ethics board approved the study.

## Results

### Baseline characteristics

Out of 6416 patients with AMI who underwent revascularization, 2.8% were Chinese, 8% were South Asian and 89.2% were White patients. By the revascularization procedure, out of 4729 patients with AMI who underwent PCI, 3% were Chinese, 7.8% were South Asian and 89.2% were White patients. Furthermore, 1687 patients underwent CABG within one year of AMI out of which 2.6% were Chinese, 8.1% South Asian and 89.3% were White patients. Among those who underwent PCI (Table 1), South Asian patients were younger at the time of AMI presentation compared to Chinese and White patients. Diabetes mellitus was more prevalent among South Asian patients, followed by Chinese and White patients. Chinese were more likely to have hypertension compared to their South Asian and White counterparts. Further, the prevalence of cerebrovascular disease and cardiac dysrhythmias was highest among Chinese and lowest among South Asian patients.

**Table 1:** Characteristics of Chinese, South Asian and White patients who underwent percutaneous coronary intervention

**Characteristics**	**Chinese**	**South Asian**	**White**	***p***
**N = 4729**	**n = 139**	**n = 371**	**n = 4219**	
Female	35 (25.2)	74 (20.0)	1153 (27.3)	0.008
Age (years)				< 0.0001
< 50	18 (12.9)	70 (18.9)	593 (14.1)	
50 − 64	42 (30.2)	164 (44.2)	1587 (37.6)	
65 − 74	54 (38.9)	88 (23.7)	1152 (27.3)	
> 75	25 (18.0)	49 (13.2)	887 (21.0)	
Distance to nearest hospital ≥ 50 km	5 (3.6)	42 (11.3)	1306 (31.0)	< 0.0001
Income quintile*				< 0.0001
1 (low)	39 (28.3)	85 (23.22)	902 (22.5)	
2	34 (24.6)	106 (29.0)	830 (20.7)	
3	25 (18.1)	85 (23.2)	771 (19.3)	
4	15 (10.9)	52 (14.2)	774 (19.4)	
5 (high)	25 (18.1)	38 (10.4)	724 (18.1)	
Diabetes mellitus	26 (18.7)	92 (24.8)	641 (15.2)	< 0.0001
Heart failure	18 (13.0)	30 (8.1)	330 (7.8)	0.09
Hypertension	52 (37.4)	92 (24.8)	1041 (24.7)	0.003
Cerebrovascular disease	5 (3.6)	0 (0.0)	43 (1.0)	0.0015
Renal disease	5 (3.6)	5 (1.4)	63 (1.5)	0.13
Cardiogenic shock	3 (2.2)	4 (1.1)	44 (1.0)	0.46
Cardiac dysrhythmia	18 (13.0)	24 (6.5)	431 (10.2)	0.04
Severity of coronary disease†				0.6
Single-vessel	51 (37.8)	135 (37.1)	1481 (36.2)	
2-vessel	39 (28.9)	119 (32.7)	1341 (32.8)	
3-vessel	38 (28.1)	98 (26.9)	1126 (27.5)	
Left main	7 (5.2)	9 (2.5)	130 (3.2)	
Normal	0 (0)	3 (0.8)	12 (0.3)	

Variables presented as n (%); Analyses: comparing categories across ethnic groups using a Chi-square test (2-*df*). *Data are missing for 224 patients (Chinese 0.7%, South Asians 1.3%, and White 5.2%). ^†^Data are missing for 140 patients (Chinese 2.9%, South Asians 1.9%, White 3.1%).

As outlined in Table 2, similar to the PCI population, among patients who underwent CABG, South Asian patients were younger at AMI presentation and more likely to have diabetes mellitus compared to their Chinese and White counterparts. However, the prevalence of cardiogenic shock was highest among Chinese patients, followed by South Asian and White patients. The highest prevalence of 3-vessel coronary artery disease was among South Asians, followed by White and Chinese patients.

**Table 2:** Characteristics of Chinese, South Asian and White patients who underwent coronary artery bypass grafting surgery within one year of AMI

**Characteristics**	**Chinese**	**South Asian**	**White**	***p*** **value**
**N = 1687**	**n = 43**	**n =137**	**n =1507**	
Female	8 (18.6)	25 (18.3)	319 (21.2)	0.68
Age (years)				0.0717
< 50	3 (7.0)	15 (11.0)	111 (7.4)	
50 − 64	16 (37.2)	62 (45.2)	518 (34.4)	
65 − 74	16 (37.2)	40 (29.2)	545 (36.1)	
> 75	8 (18.6)	20 (14.6)	333 (22.10)	
Distance to the nearest hospital ≥ 50 km	2 (4.7)	15 (11.0)	514 (34.1)	< 0.0001
Income quintile*				0.52
1 (low)	11 (26.8)	38 (28.6)	328 (23.0)	
2	9 (22.0)	34 (25.6)	289 (20.3)	
3	7 (17.1)	23 (17.3)	259 (18.2)	
4	8 (19.5)	20 (15.0)	275 (19.3)	
5 (high)	6 (14.6)	18 (13.5)	273 (19.2)	
Diabetes mellitus	12 (27.9)	44 (32.1)	321 (21.3)	0.0098
Heart failure	10 (23.3)	22 (16.1)	233 (15.5)	0.38
Hypertension	11 (25.6)	47 (34.3)	423 (28.1)	0.27
Cerebrovascular disease	1 (2.3)	0 (0.0)	39 (2.6)	0.16
Renal disease	0 (0.0)	6 (4.4)	35 (2.3)	0.19
Cardiogenic shock	2 (4.7)	5 (3.7)	19 (1.3)	0.0231
Cardiac dysrrhythmia	6 (14.0)	19 (13.9)	208 (13.8)	0.99
Severity of coronary disease†				0.49
Single vessel	1 (2.5)	6 (4.8)	73 (5.3)	
2 vessel	8 (19.5)	14 (11.2)	229 (16.8)	
3 vessel	21 (51.2)	85 (68.0)	795 (58.4)	
Left main	11 (26.8)	20 (16.0)	264 (19.4)	
Normal	0 (0.0)	0 (0.0)	1 (0.1)	

Variables presented as n (%); Analyses: comparing categories across ethnic groups using a Chi-square test (2-*df*). *Data are missing for 89 patients (Chinese = 4.7%, South Asians 3%, and White 5.5.%).

^†^Data are missing for 159 patients (Chinese 4.7%, South Asians 8.8%, White 9.6%).

### Outcomes

One hundred and sixteen patients died within 30 days from PCI (6.5% of Chinese patients (n = 9), 3.0% of South Asian (n = 11), and 2.3% of White patients (n = 96)). Within a year from PCI, there were 23, 16, and 23 fatalities per 1000-patient years in Chinese, South Asian and White patients, respectively. Among those who underwent CABG within 30 days following AMI, 4.7% of Chinese (n = 2), 2.2% of South Asians (n = 3), and 4.1% of White patients (n = 62) died. With regards to long-term mortality following CABG, there were 0, 30 and 21 events per 1000-patient years in Chinese, South Asian and White patients, respectively. After controlling for baseline characteristics, the odds of short-term mortality were significantly higher among Chinese compared to White patients who underwent PCI (Table 3). In the adjusted analyses, the risk of recurrent AMI and heart failure was found to be higher among South Asian than in White patients following PCI. No other ethnic differences were noted among patients who underwent PCI. Among patients who underwent CABG, there were no statistically significant ethnic differences in mortality and risk of recurrent AMI, however, the risk of heart failure was significantly higher among South Asian patients than in White patients.

**Table 3:** Ethnic differences in outcomes among patients who underwent PCI and CABG

**Outcome**	**Chinese vs. White**	**South Asians vs. White**	**Chinese vs. South Asian**
PCI			
30-day mortality OR (95% CI) [*p*]	2.36 (1.12-5.00) [p = 0.02]	1.63 (0.83-3.20) [p = 0.15]	1.45 (0.56-3.77) [p = 0.45]
1-year mortality HR (95% CI) [*p*]	0.69 (0.30-1.57) [p = 0.37]	0.77 (0.43-1.40) [p = 0.39]	0.89 (0.33-2.40) [p = 0.82]
Recurrent AMI* HR (95% CI) [*p*]	1.04 (0.71-1.51) [p = 0.86]	1.34 (1.08-1.67) [p = 0.007]	0.77 (0.51-1.17) [p = 0.22]
Heart failure* HR (95% CI) [*p*]	1.72 (0.74-4.02) [p = 0.21]	1.81 (1.00-3.29) [p = 0.051]	0.95 (0.36-2.55) [p = 0.92]
CABG			
30-day mortality OR (95% CI) [*p*]	0.91 (0.20-4.22), [p = 0.91]	0.64 (0.20-2.01) [p = 0.44]	1.43 (0.23-9.04) [p = 0.70]
1-year mortality HR (95% CI) [*p*]	0.14 (0.001-1.05) [p = 0.18]	1.12 (0.50-2.25) [p = 0.76]	0.13 (0.01-2.43) [p = 0.17]
Recurrent AMI** HR (95% CI) [*p*]	0.52 (0.07-3.80) [p = 0.52]	0.44 (0.10-1.85) [p = 0.26]	1.18 (0.11-13.24) [p = 0.90]
Heart failure** HR (95% CI) [*p*]	1.72 (0.52-5.75) [p = 0.38]	2.06 (0.92-4.61) [p = 0.08]	0.84(0.21-3.32) [p = 0.80]

Models are adjusted for age, sex, SES, distance to nearest hospital, time from diagnosis of AMI to a procedure (PCI/CABG), severity of coronary disease and comorbid conditions.

PCI: Chinese, n = 139; South Asians, n = 371; White, n = 4219.

CABG: Chinese, n = 43; South Asians, n = 137; White, n = 1507.

*among survivors only, Chinese, n = 123; South Asian, n = 347; White, n = 3879.

**among survivors only, Chinese, n = 31; South Asian, n = 85; White, n = 1010.

## Discussion

In this observational study we found significantly worse prognosis in South Asian and Chinese patients with AMI following revascularization compared to their White counterparts. These observed ethnic differences in mortality, risk of recurrent AMI, and heart failure were independent of age, sex, socio-economic status, distance to the nearest hospital, the duration from an AMI diagnosis to a revascularization procedure, severity of coronary disease and comorbid conditions.

Our findings of higher short-term mortality among Chinese patients following PCI are in contrast to those of a recent study where the rates of all-cause and cardiac mortality following PCI were lower in Asian patients (Singapore, Hong Kong, and Malaysia) compared to their counterparts from Western Europe [9]. This difference may be due to the fact that our population was an AMI population compared to a lower risk unselected coronary disease population in the study above. Moreover, we directly compared outcomes in ethnic groups within a single health care system whereas Klomp et al. indirectly compared patients in differing health care systems [9,26].

Higher short-term mortality following PCI among Chinese patients compared with their White counterparts may have occurred for several reasons. Chinese patients may have had arrived later to the emergency department [27,28] resulting in less effectiveness of the procedures or more extensive myocardial damage at time of PCI [29]. However, in our study, we extensively adjusted for differences in baseline prognostic characteristics and extent of coronary disease. Chinese patients may also have had higher post procedural complications or differences in coronary artery disease management. Chinese patients have been shown to have a lower rate of filling their prescriptions and for adhering to secondary prevention medications [30,31]. Furthermore, they have a greater use of herbal medications [32]. In addition, there may be genetic differences in antiplatelet responsiveness between the ethnic groups [33,34] placing Chinese patients at higher risk of peri- and post procedural complications.

While no difference between South Asian and White patients was observed for mortality, South Asian patients were found to be at greater risk of non-fatal adverse cardiovascular events following PCI and CABG compared with White patients, even when examining survivors only. Other studies examining ethnic differences in outcomes following PCI or CABG have reported conflicting results. While several studies of unselected coronary disease patients have revealed no difference in fatal cardiac outcomes between South Asian and White patients following PCI [7,8] and CABG [13], others have observed higher CABG postoperative mortality in South Asian than in White patients [10-12]. However, the latter studies did not adjust for prognostic factors that may have contributed to the conflicting results and did not directly study AMI populations.

Our study represents a direct comparison of ethnic differences of AMI patients in a single health care system using extensive adjustment for prognostic factors including severity of coronary disease. However, several study limitations should be noted. First, this study is an observational study and the ideal study design for determining efficacy of these procedures following AMI would be a randomized trial. This study is thus susceptible to treatment-selection bias and bias from residual confounding. Although we did adjust for multiple potential confounding factors, we were not able to control for risk behaviors (such as smoking, physical activity or diet), dyslipidemia or extent of infarct (non-ST elevation MI, or peak cardiac enzyme level) that could explain the observed difference in outcomes. Second, ethnicity was defined using surname algorithms because self-reported ethnicity data were not available. However, surname algorithms have been shown to have high sensitivity and specificity; however, there is a possibility that some of the ethnic categories were misclassified that could potentially underestimate inter-ethnic differences in study outcomes. Additionally, we did not know generational status of patients, but likely, given the age of patients in the cohort, most patients would be first generation immigrants.

## Conclusions

This observational study found a worse prognosis in South Asian and Chinese patients following revascularization compared to their White counterparts. Observed ethnic disparities in outcomes were present despite adjustment for demographic, socio-economic and clinical characteristics. We believe that the availability of interpretation and translation services, the presence of education programs teaching the importance of adherence to secondary prevention medication, the recognition of early signs of AMI and timely seek of care, along with equitable access to health care, may help reduce ethnic disparities in postrevascularization outcomes. In addition, given the inter-ethnic differences in the response to clopidogrel antiplatelet therapy, a future personalized approach to therapy may further reduce the disparity in postrevascularization outcomes.

## Competing interests

The authors declare no competing interests.

## Authors’ contributions

DG participated in the design of the study, interpreted the data and drafted the manuscript. NAK made substantial contributions to design of the study and data interpretation, helped to draft the manuscript, and she critically revised it. HQian performed statistical analyses and contributed to data interpretation. SK participated in the discussion and critically revised the manuscript. GS contributed to the conception of the study, participated in the discussion and critically revised the manuscript. HQuan participated in the discussion and critically revised the manuscript. MHM participated in the discussion and critically revised the manuscript. BJO participated in the discussion and critically revised the manuscript. AFA conceived the study, participated in its design, data interpretation, the manuscript discussion and critically revised the manuscript. All authors read and approved the final manuscript.

## Pre-publication history

The pre-publication history for this paper can be accessed here:

http://www.biomedcentral.com/1471-2261/13/121/prepub
